# Effects of Production System With or Without Growth-Promoting Technologies on Growth and Blood Expression of (Cyto)Chemokines and Heat Shock and Tight Junction Proteins in *Bos taurus* and *indicus* Breeds During Summer Season

**DOI:** 10.3390/vetsci12010065

**Published:** 2025-01-16

**Authors:** Mark Branine, Ashley K. Schilling-Hazlett, Pedro H. V. Carvalho, Kim R. Stackhouse-Lawson, Edilane C. Martins, Julia T. da Silva, Laura Amundson, Chris Ashworth, Mike Socha, Sami Dridi

**Affiliations:** 1 Zinpro Corporation, Eden Prairie, MN 55344, USA; mbranine@zinpro.com (M.B.); lamundson@zinpro.com (L.A.); cashworth@zinpro.com (C.A.); msocha@zinpro.com (M.S.); 2 AgNext, Colorado State University, Fort Collins, CO 80523, USA; ashley.schilling@colostate.edu (A.K.S.-H.); pedro.carvalho@colostate.edu (P.H.V.C.); kim.stackhouse-lawson@colostate.edu (K.R.S.-L.); julia.travassosdasilva@colostate.edu (J.T.d.S.); 3 Center of Excellence for Poultry Science, University of Arkansas, 1260 W. Maple Street, Fayetteville, AR 72701, USA

**Keywords:** cattle breed, heat stress, blood, gene expression, growth

## Abstract

Heat stress (HS) induced by global warming is a real welfare, productivity, and economic burden of cattle production. However, some cattle breeds have superior physiological adaptive traits to others, yet the underlying molecular mechanisms are not fully defined. The present study aimed, therefore, to determine the expression profile of stress-related molecular signatures in the blood of thermosensitive Angus (*Bos taurus*) and thermotolerant Brahman (*Bos indicus*) cattle breeds managed without (CON) or with growth-promoting technology (TRT) during the summer (April–October, 2023) season in Colorado, US. Body weight (BW) was significantly increased from April to October, and the amplitude was greater for the Angus compared to the Brahman breed. The TRT system slightly increased BW, mainly in the Angus breed. Molecular analyses showed that all tested genes were expressed in beef cattle blood. When comparing production systems, the expression of HSP1A1 was significantly upregulated, and HSP90 was downregulated in CON compared to TRT cattle. The expression of IL6, CCL20, and OCLN was induced by the CON system only in the Angus and not in the Brahman breed. At the breed level, Angus cattle exhibited greater expression of IL10, CCL20, and CLDN1 compared to their Brahman counterparts. There was a significant period by production system as well as period by breed interactions. The expression of HSP1A1 increased in both breeds during October. The expression of IL10, CXCL14, CXCR2, and CLDN1 was affected by the production systems in a period-dependent manner. However, the expression of IL6, CXCL14, CCL5, and CXCR2 was upregulated in Angus cattle in a period-sensitive manner. In summary, HSPs, (chemo)cytokines, and tight junction proteins are expressed in the whole blood of beef cattle, and their expression is regulated in a breed-, period-, and/or production system-dependent manner. This could open new vistas for future research to identify molecular signatures for non-invasive stress monitoring and/or marker-assisted genetic selection for robustness and resilience to HS.

## Simple Summary

Although the beef cattle industry supports the livelihoods and food security of billions of people worldwide, it is facing several challenges, including the need to adapt to global warming. Angus breeds have been reported to have a better growth rate compared to Brahman breeds, but are more sensitive to environmental temperatures, yet the underlying physiological and molecular mechanisms are not fully defined. We aimed here to determine the effect of the summer season (April–October 2023) on the expression of heat shock proteins, cytokines, chemokines, and tight junction proteins in the blood of Angus and Brahman breeds reared under two production systems (with or without growth-promoting technology, TRT). As expected, Angus cattle had better body and hot carcass weights than Brahman cattle. The TRT system improved growth performance, particularly in Angus cattle. The expression of HSPs, cytokines, chemokines, and tight junction proteins was breed-, system-, and/or period-dependent. In total, this study provided new insights related to the regulation of the abovementioned molecular markers that can be used to develop non-invasive methods to monitor stress in beef cattle and/or for marker-assisted genetic selection.

## 1. Introduction

Unusually warm and longer seasonal temperatures with large, abrupt, and widespread heat waves have occurred over the past three decades [1,2,3]. Predicted global warming will markedly increase and be even larger, more intense, and more frequent in the next century [4,5,6,7]. Global warming-induced heat stress (HS) and droughts are already affecting animals, insects, and crops [8], and, thereby, threatening agriculture production sustainability [9,10].

With a global production of over 59 million metric tons [11], the beef cattle industry supports the livelihood and food security of millions, if not billions, of people worldwide. The United States (US) is the world’s leading producer of beef, and the US beef cattle industry is the largest fed-cattle industry in the world [11,12]. As the global human population is predicted to grow to 9–10 billion people by 2050, the Agriculture Organization of the United Nations (FAO) estimated that animal-based protein production will have to increase by 73% to feed these future people and fulfill and meet their nutritional needs [13]. This will be very challenging, mainly under the current and projected planetary boundaries, with limited, even scarce, natural resources such as land, water, and energy [14,15,16,17,18,19].

As cattle are reared in different production systems, whether grazing, confinement, or other, US beef production, which contains a wide variety of breeds and crosses (Angus, Hereford, Simmental, Charolais, Gelbvieh, Brangus, Limousin, Beefmaster, Shorthorn, Brahman), is (in)directly and constantly exposed to natural climatic conditions, including heat or cold stress, which can influence its productivity and sustainability [12]. The adverse effects of HS on feed intake, production losses, welfare, and mortality are well documented [20,21,22,23,24,25]. The amplitude of these negative effects depends on beef breeds and strains. For instance, Angus steers have a faster growth rate and better feed efficiency (conversion of feed into meat) but are more susceptible and vulnerable to HS compared to Brahmans [26,27], suggesting that they are not as well adapted to hot conditions.

At the organismal levels, the effects of HS can extend from discomfort to systemic and organ damage, and, under extreme events, to mortality. During the past few years, periodic extremes of heat in the major cattle-feeding areas of the United States have resulted in the deaths of many thousands of animals, resulting in significant animal wellbeing and welfare issues as well as major economic losses. Depending on the type, source, severity, and duration of the stress, organisms (cells) can develop complex and highly efficient stress responses and protein quality control systems to ensure their survival or activate cell-death pathways. These responses are controlled by a complex molecular network, which includes heat shock proteins, which are ubiquitously expressed molecular chaperones, and are the first line of cellular defense against HS [28]. Heat stress also modulates the expression of (anti)pro-inflammatory cytokines and chemokines, which play pivotal roles in maintaining immune and cellular homeostasis [29,30]. Heat stress can affect tight junction proteins, leading to altered cell-to-cell communication and permeability, and thereby alters the immune response [31]. These molecular markers and their regulation by HS are still not well defined in bovine species, and we hypothesize that they might differ between the thermosensitive (Angus) and thermotolerant (Brahman). The present study aimed, therefore, to assess the expression profile of these molecular signatures in the blood of Angus and Brahman beef cattle reared under two production systems (with and without production technologies) during the summer of 2023 (April to October) season for subsequent identification of markers for non-invasive monitoring of stress and/or the development of marker-assisted genetic selection for robustness and HS resilience.

## 2. Materials and Methods

### 2.1. Ethical Statement

This study was approved by the Institutional Animal Care and Use Committee at the Colorado State University (protocol number 3712-13). This experiment was carried out at the Animal Research Farm at AgNext, (Colorado State University, Fort Collins, CO, USA), during the summer season, from April to October 2023.

### 2.2. Cattle Breeds and Experimental Design

**Figure 1:**
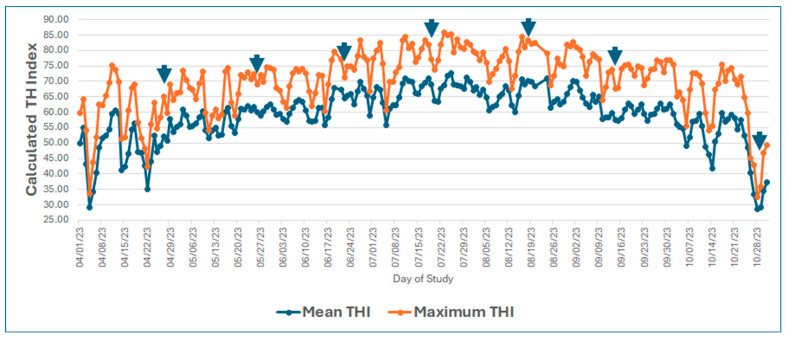
Daily mean and maximum temperature–humidity index (THI) values during the study. The THI was calculated using the mean or maximum daily temperature and relative humidity value. Arrows denote days when samples were collected.

Steers from two cattle breeds, Angus (*Bos taurus*, n = 100) and Brahman (*Bos indicus,* n = 100), were received at the Colorado State University’s Agricultural Research, Development and Education Center (ARDEC) research feedlot, located approximately 16 km north of Fort Collins, CO. Angus cattle were procured from one ranch located in Montana. Brahman cattle were procured from a ranch in Texas. Both groups of cattle were shipped from their respective places of origin to the ARDEC and were received in April 2023. Upon arrival, all cattle were allowed to rest and provided with long-stem hay and free access to water. On 27 and 28 April 2023 (d − 1 and d 0), steers were individually weighed to obtain initial body weight (iBW) and randomized to two production systems within each breed, as well as assigned to blocks based on their iBW. The technology production system (TRT) consisted of administering to both Angus and Brahman steers growth-promoting technologies commonly used in commercial beef feedlots. Specifically, these included providing an initial anabolic in-ear implant on d 0 (100 mg trenbolone acetate/14 mg estradiol benzoate; Synovex Choice^®^, Zoetis, Parsippany, NJ, USA) with a reimplant on d 84 (200 mg trenbolone acetate/28 mg estradiol benzoate; Synovex Plus^®^, Zoetis) 96 days prior to slaughter. In the growing and finishing diets), cattle were fed 35 g monensin/ton DM basis (Rumensin^®^, Elanco, Greenfield, IN, USA) and 7 g tylosin/ton DM basis (Tylan^®^, Elanco). Approximately 42 days prior to slaughter, cattle were fed 27 g ractopamine hydrochloride (RAC)/ton DM basis (Actogain^®^; Zoetis Animal Health), allowing for a 2d withdrawal period prior to harvest. The second production system consisted of providing Angus and Brahman steers with no growth-promoting technologies and was designated as a non-technology or control (CON) feeding program. At initial processing, in addition to being individually weighed, all cattle received a common vaccination regimen, including bovine rhinotracheitis virus–diarrhea–parainfluenza 3–respiratory syncytial virus vaccine (Bovi-Shield GOLD^®^ 5, Zoetis, Kalamazoo, MI, USA) and a clostridial diseases vaccine (Ultrachoice^®^ 8, Zoetis, Kalamazoo, MI, USA). Steers were administered medication for internal and external parasites (Dectomax^®^, Zoetis, Kalamazoo, MI, USA) and were orally drenched with albendazole (Valbazen^®^, Zoetis, Kalamazoo, MI, USA). Both a visual and radio frequency identification tag were administered to provide a means for individual animal identification. Following randomization, all steers were housed by block (5 blocks/production system) in 10-head research feedlot pens (4 pens/block) for the first 84 days of the study. After day 84, pens were combined by production system and breed and assigned to climate-smart research pens (50 steers/pen/breed) for the remainder of the feeding period. These pens were designed to measure individual animal feed intake on a daily basis as well as an estimate of the daily amount of methane output per animal (C-Locke Inc; Rapid City, SD, USA). All cattle were weighed and shipped to the JBS facility in Greeley, CO, on d 180, where individual carcass data were collected. The temperature–relative humidity index (THI) was determined during the study period and is summarized in Figure 1.

Individual animal BW was collected at approximately 28-day intervals throughout the study. At each weighing, three steers/pen were randomly selected for the collection of blood. Whole blood samples from the same steers were collected by jugular venipuncture, at each weighing, with 60 animals representing each breed and production system (15/group). Blood samples were collected into heparinized tubes and kept on ice until they were aliquoted with Trizol LS (Life Technologies, Carlsbad, CA, USA) for total RNA isolation and stored at −80 °C until further analysis. From the 60 animals from which whole blood was collected, 12 steers were randomly selected, per breed × production system (24 total), for further gene expression analysis.

### 2.3. RNA Extraction, Reverse Transcription, and Real-Time Quantitative PCR

Total RNA was extracted using Trizol LS reagent (Life Technologies, Carlsbad, CA, USA) according to the manufacturer’s recommendations. RNA integrity and quality were assessed using 1% agarose gel electrophoresis, and the RNA concentrations and purity were determined for each sample by Take 3 Micro-Volume Plates using a Synergy HT multi-mode micro plate reader (BioTek, Winooski, VT, USA). The RNA samples were RQ1 RNase-free DNase-treated (Promega, Madison, WI, USA), and RNA (1 µg) was reverse transcribed using a qScript cDNA Synthesis Kit (Quanta Biosciences, Gaithersburg, MD, USA) in a 20 µL total reaction. The reverse transcription reaction was performed at 42 °C for 30 min, followed by an incubation at 85 °C for 5 min. Real-time quantitative PCR (Applied Biosystems 7500 Real-Time PCR system) was performed using 5 µL of 10× diluted cDNA, 0.5 µM of each forward and reverse specific primer, and SYBR Green Master Mix (ThermoFisher Scientific, Rockford, IL, USA) in a 20 µL total reaction, as previously described by Dridi et al. [32]. Oligonucleotide primers specific for cattle heat shock proteins (HSP60, HSP1A1, HSP90), interleukins (IL-1β, IL6, IL10, IL18), tumor necrosis factor alpha (TNFα), C-reactive protein (CRP), X-C motif chemokine ligand 1 (XCL1), C-X-C motif chemokine ligands (CXCL12 and 14), C-C motif chemokine ligands (CCL2, CCL4, CCL5, CCL20), C-C motif chemokine receptor 2 (CCR2), C-X-C motif chemokine receptors (CXCR1 and 2), claudin 1 (CLDN1), occludin (OCLN), and ribosomal 18S as a housekeeping gene are summarized in Table 1. Relative expressions of target genes were determined by the 2^−∆∆Ct^ method [33]. The Brahman steers, with the technology production system, in the April period, were used as calibrators.

### 2.4. Statistical Analysis

Growth performance (body weight) and gene expression data were analyzed by three-way repeated measures ANOVA (breed × production system × period and their interactions). If ANOVA revealed significant effects, the means were compared by Tukey’s HSD multiple comparison test using Graph Pad Prism version 9.00 for Windows (Graph Pad Software, La Jolla, CA, USA). If the interactions were not significant, the main factors were analyzed separately using two-way ANOVA, one-way ANOVA, or Student’s “t” as appropriate. Data are presented as the mean ± the SEM, and the statistical significance was set at *p* < 0.05.

## 3. Results

### 3.1. Growth Performance (Body Weight, Body Weight Gain, and Hot Carcass Weight)

For body weight (BW), there was no significant period by breed by production system interaction, nor period by production system interaction; however, the period × breed interaction was significant (*p* = 0.0424; Table 2). Body weight significantly increased from April to October, and the amplitude was greater for the Angus compared to the Brahman breed (Table 2). The production system TRT increased BW mainly in the Angus breed (Table 2). Similarly, TRT-treated Angus steers had a better growth rate, mainly during June and October (Table 3), and significantly higher HCW at the end of the experiment (Table 4).

### 3.2. HSP Gene Expression Profile

**Figure 2:**
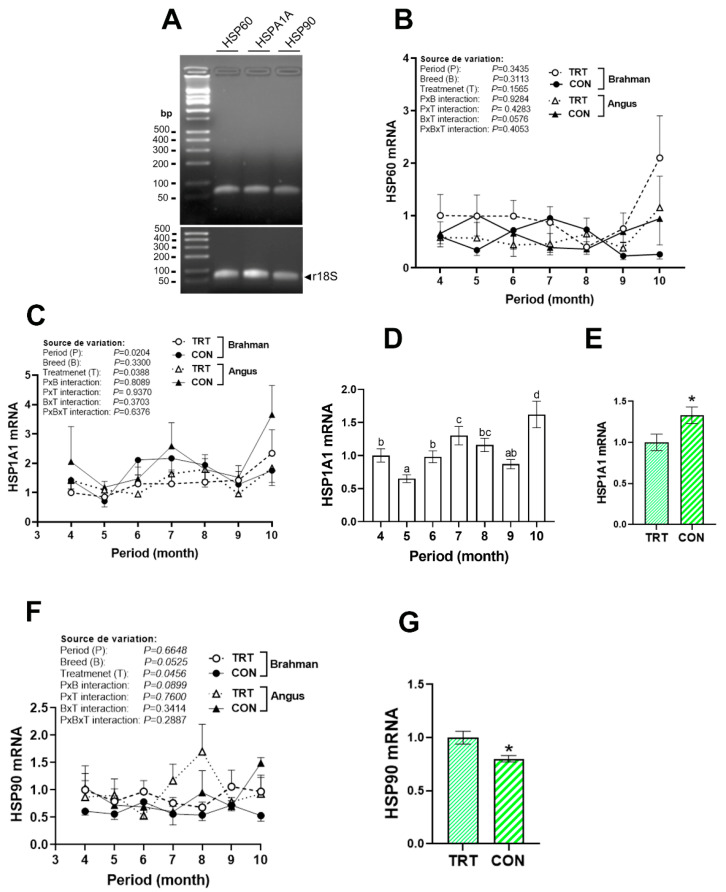
Circulating expression of HSPs in Angus and Brahman breeds subjected to two management systems during summer season. mRNA levels of HSP60 (**A**,**B**), HSP1A1 (**A**,**C**–**E**), and HSP90 (**F**,**G**) were determined by qPCR using 2^−ΔΔCT^ method (33), r18S as a housekeeping gene, and Brahman–CON–April as a calibrator. If the breed × treatment × period interaction was not significant, the main effects were analyzed separately by one-way ANOVA or *t*-test as appropriate. For the period, breed, or treatment effect, April, Brahman, or CON was used as a control. CON, control; HSP, heat shock protein; TRT, technology production system. * indicates a significant difference at *p* < 0.05. Different lowercase letters indicate significant differences at *p* < 0.05.

**Figure 3:**
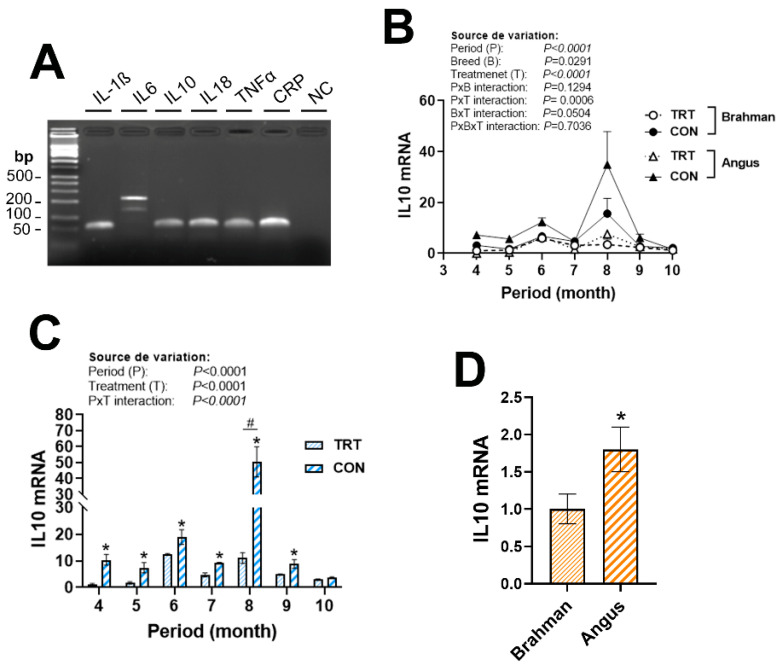
Circulating expression of anti-inflammatory IL10 in Angus and Brahman breeds subjected to two management systems during summer season. All measured cytokines are expressed in beef cattle blood (**A**). The expression of IL10 gene (**B**–**D**) was determined by qPCR, as described in Materials and Methods Section. Brahman breed, CON, and April period was used as a control and calibrator. CON, control; IL-10, interleukin 10; TRT, technology production system. * and # indicate a significant difference at *p* < 0.05. Different lowercase letters indicate significant differences at *p* < 0.05.

All tested genes (HSPs, cytokines, chemokines, and tight junction proteins) were expressed in beef cattle blood (Figures 2A, 3A, 5A, and 8A). Although HSP60 gene expression remained unchanged in all groups (Figure 2B), HSPA1A was upregulated, and HSP90 was downregulated in the CON cattle compared to their TRT counterparts (Figure 2C–G). Circulating HSPA1A gene expression fluctuated during the summer season, with a decreased expression in May, and increased levels in July and October (Figure 2D).

### 3.3. Gene Expression Profile of (Anti)Pro-Inflammatory Cytokines

**Figure 4:**
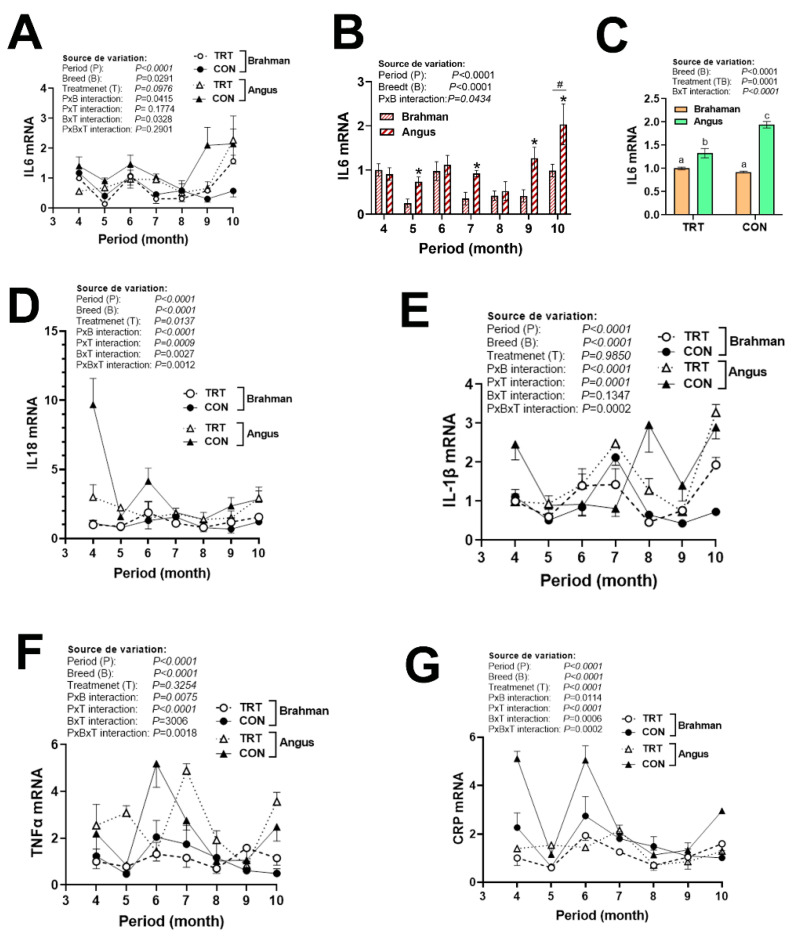
Circulating expression of pro-inflammatory cytokines in Angus and Brahman breeds subjected to two management systems during summer season. The expression of IL6 (**A**–**C**), IL18 (**D**,**E**), TNFα (**F**), and CRP (**G**) was measured by qPCR. Brahman breed, CON, and April period was used as a control and calibrator. CON, control; CRP, C-reactive protein; IL, interleukin; TNFα, tumor necrosis factor alpha; TRT, technology production system. * and # indicate a significant difference at *p* < 0.05. Different lowercase letters indicate significant differences at *p* < 0.05.

Circulating cytokine expression was affected by period, breed, and/or production system. The expression of IL10 was greater in Angus compared to Brahman cattle (Figure 3D) and was induced by the CON production system during the study period (Figure 3C). The expression of IL6 was greater in Angus compared to Brahman cattle during May, July, September, and October (Figure 4A,B), and was induced by the CON production system mainly in Angus, but not in Brahman cattle (Figure 4C). A significant period × breed × production system interaction was discerned for IL18, IL-1β, TNFα, and CRP (Figure 4D–G). Abundances of IL18 mRNA were greater in CON Angus cattle during April and decreased for the rest of the summer season. The expression of IL-1β, however, was triphasic, with greater levels in CON Angus cattle during April, August, and October (Figure 4E). The expression of TNFα was upregulated in CON Angus cattle during June, and in TRT Angus cattle during July (Figure 4F). CRP mRNA levels were induced by the non-technology production system in both breeds in April and June, but the amplitude was greater in the Angus compared to the Brahman cattle (Figure 4G).

### 3.4. Gene Expression Profile of Chemokine Ligands and Receptors

**Figure 5:**
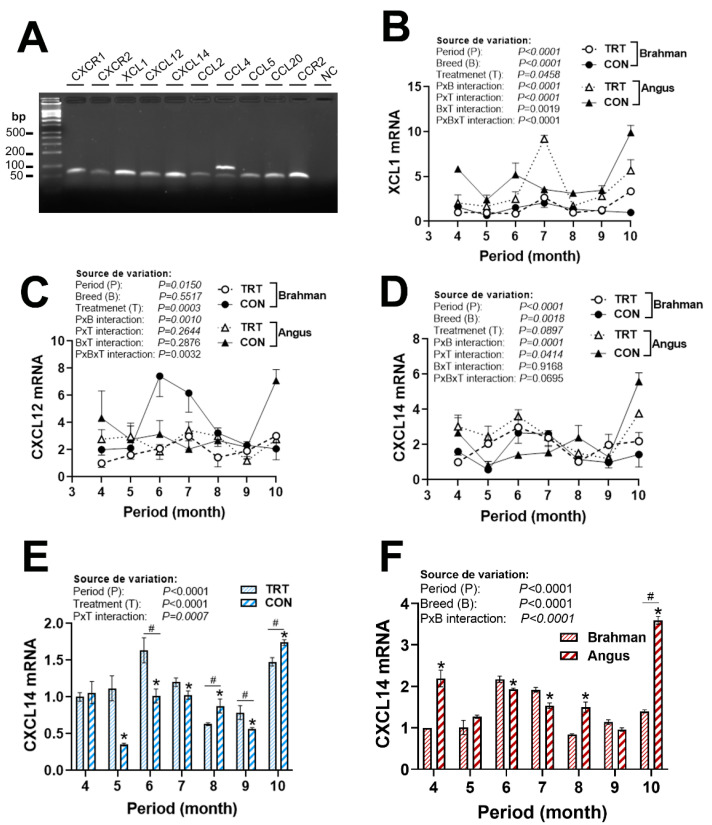
Circulating expression of C-X-C and X-C motif chemokine ligands in Angus and Brahman breeds subjected to two management systems during summer season. All tested chemokines were expressed in blood (**A**). The expression of XCL1 (**B**), CXCL12 (**C**), and CXCL14 (**D**–**F**) was determined by qPCR. Brahman breed, CON, and April period was used as a control and calibrator. CON, control; CXCL, C-X-C motif chemokine ligand; TRT, technology production system; XCL, X-C motif chemokine ligand. * and # indicate significant differences at *p* < 0.05 between periods and treatments, respectively.

**Figure 6:**
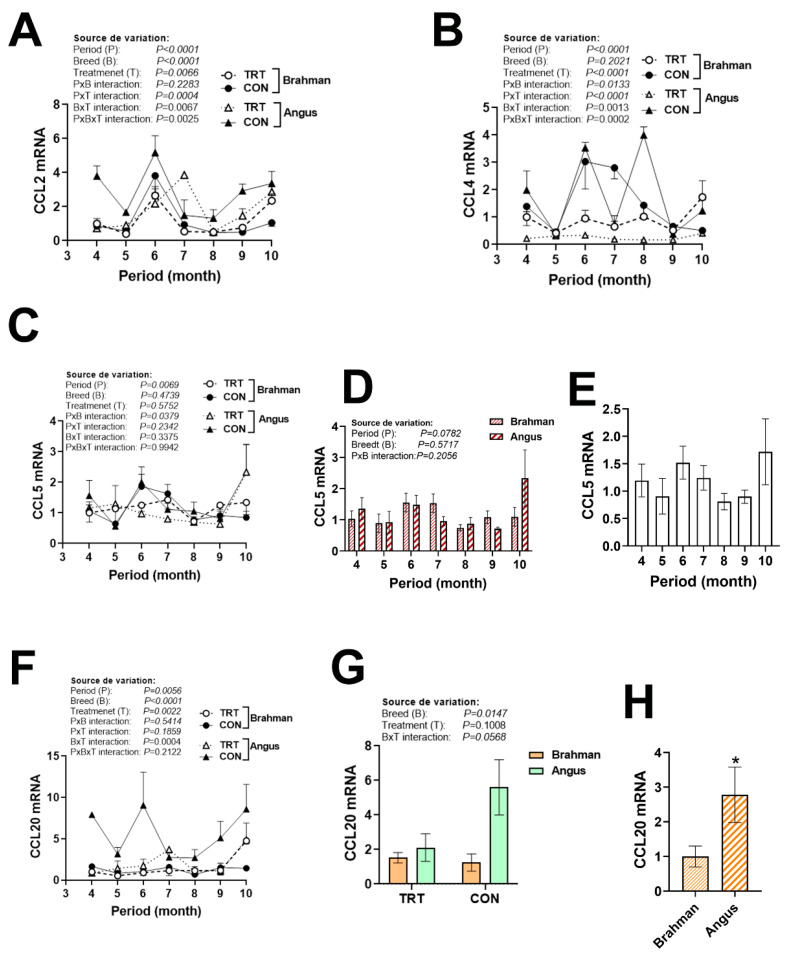
Circulating expression of C-C motif chemokine ligand in Angus and Brahman breeds subjected to two management systems during summer season. The expression of CCL2 (**A**), CCL4 (**B**), CCL5 (**C**–**E**), and CCL20 (**F**–**H**) was determined by qPCR. Brahman breed, CON, and April period was used as a control and calibrator. CCL, C-C motif chemokine ligand; CON, control; TRT, technology production system. * indicates a significant difference at *p* < 0.05.

**Figure 7:**
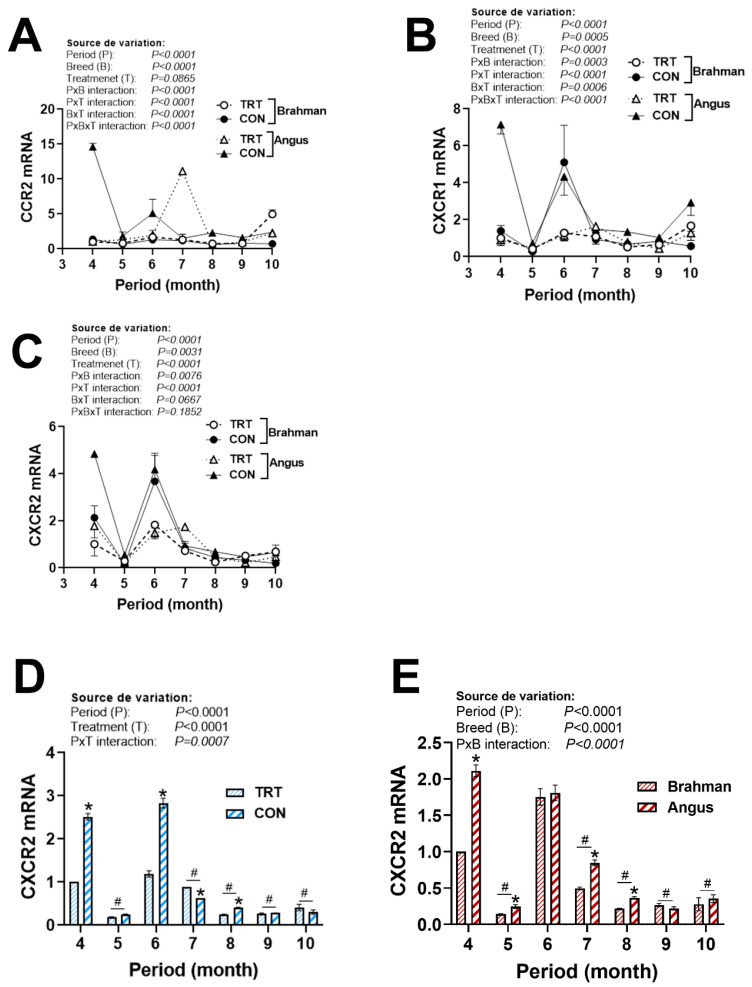
Circulating expression of C-C and C-X-C motif chemokine receptors in Angus and Brahman breeds subjected to two management systems during summer season. The expression of CCR2 (**A**), CXCR1 (**B**), and CXCR2 (**C**–**E**) was determined by qPCR. Brahman breed, CON, and April period was used as a control and calibrator. CCR2, C-C motif chemokine receptor; CON, control; CXCR, C-X-C motif chemokine receptor; TRT, technology production system. * and # indicate significant differences at *p* < 0.05 between periods and Breeds, respectively.

There were significant period × breed × production system interactions for chemokine ligands (XCL1, CXCL12, and CCL2/4, Figure 5B,C and Figure 6A,B) and chemokine receptors (CCR2 and CXCR1, Figure 7A,B), but not for CXCL14 (Figure 5D), CCL20 (Figure 6F), and CXCR2 (Figure 7D). The expression of CCL2 was greater in CON Angus cattle during April, June, September, and October (Figure 6A). CCL4 mRNA levels were induced in CON Angus cattle during June and August, and in CON Brahman cattle during June and July (Figure 6B). CCL5 expression was not affected by period or breed (Figure 6C–E). The expression of CCL20, however, was significantly greater in the Angus compared to the Brahman breed (Figure 6F–H). CXCR2 expression was upregulated by the non-technology production system in both breeds during April, June, and August, and was greater in Angus cattle during April, May, July, and August (Figure 7C–E).

### 3.5. Gene Expression Profile of Tight Junction Proteins

**Figure 8:**
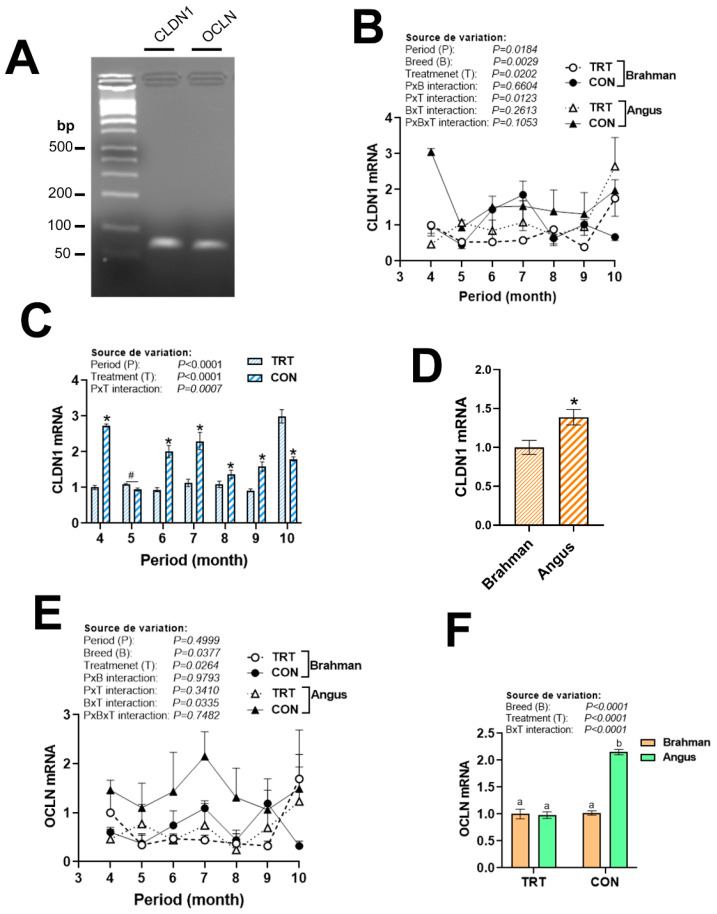
Circulating expression of tight junction protein in Angus and Brahman breeds subjected to two management systems during summer season. Both CLDN1 and OCLN were expressed in blood (**A**). The expression of CLDN1 (**A**–**D**) and OCLN (**E**,**F**) was determined by qPCR. Brahman breed, CON, and April period was used as a control and calibrator. CLDN1, claudin 1; CON, control; OCLN, occludin; TRT, technology production system. * and # indicate significant differences at *p* < 0.05 between periods and treatments, respectively. Different lowercase letters indicate significant differences at *p* < 0.05.

There were no significant period × breed × production system interactions for CLDN1 and OCLN gene expression (Figure 8B,E). The expression of CLDN1 was upregulated by the non-technology production system during April, June, July, August, and September, and then downregulated during October compared to the TRT production system (Figure 8C). CLDN1 mRNA levels were significantly greater in Angus cattle as compared to their Brahman counterparts (Figure 8D). OCLN expression was upregulated by the non-technology production system only in Angus but not in Brahman cattle (Figure 8F).

## 4. Discussion

Built on unusual years of record-breaking droughts and higher seasonal temperatures, climate simulation models predict that global warming will only intensify and exponentially rise [34]. Indeed, according to current climate models, global average temperatures are expected to increase by 2.46 to 4.10 °C by the end of this century [35]. This, in turn, will continue to adversely impact the sustainability of beef cattle worldwide. Some beef cattle breeds and strains withstand and cope better with hot environmental temperatures than others due to their superior thermoregulation ability and physiological and cellular adaptive traits, which are not fully defined. Here, we aimed to assess the expression of several potential circulating molecular signatures involved in HS responses between more heat-sensitive (Angus) and more heat-tolerant (Brahman) cattle during the summer season in Colorado, US.

As expected, and in agreement with previous studies [36,37], Angus cattle had an increased BW and growth rate compared to Brahmans. Administration of growth-promoting technologies in the form of implants, RAC, and monensin/tylosin increased the BW and daily growth rate in both breeds, although the relative magnitude of this response was greater for Angus compared to Brahman cattle. For CON cattle, again, Angus cattle were heavier and had a greater daily growth rate compared to Brahman cattle. These data are not surprising as Angus cattle have been selectively bred for an increased growth rate with the use of growth-promoting technologies. Because both anabolic implants and the β-agonist RAC have systemic effects that enhance muscle accretion and mass via hypertrophy, as well as repartitioning nutrients to support these effects and promote growth, it is interesting to speculate on how this may interact with the expression of the genes measured in this study [38,39,40,41].

Interestingly, molecular analyses showed that all tested markers, HSPs, cytokines, chemokines, and tight junction proteins (CLDN1 and OCLN), are present in beef cattle blood; however, in what specific cells (red or white blood cells, leukocytes, monocytes, PBMCs, etc.) they reside warrants further investigations. Heat shock proteins 60 and 70 have previously been reported to be expressed in cattle PBMCs and affected by HS [42]. Cytokines, chemokines, and their related receptors were also found in whole blood from BRD calves [43]. Although there is not much information on cattle blood cells, tight junction proteins, claudins, and occludin were found in human circulation and cerebrospinal fluid (CSF) [44].

Although the expression of HSP60 and HSP90 was not affected in our experimental conditions, HSPA1A (HSP70) was upregulated during sampling days in July (22nd) and October (28th), when the maximum temperature–humidity index (THI) was 81.99 and 32.62, respectively. During the days before sampling, the THIs were 76.07 (July 21st) and 42.91 (October 27th), which suggests that blood HSP70 was more responsive to THI fluctuations than HSP60 and HSP90, corroborating a previous study [45]. Of particular interest, despite the fact that most studies reported an upregulation of HSPs [46,47], the technology (RAC) production system in our experimental conditions significantly decreased the blood level of HSP60 and induced that of HSP90, suggesting potential different roles of these two HSP families. In addition to responses to stress insults, these proteins play roles in cell signaling, energy store mobilization, and cell oxygenation, to mention a few [48].

The increased expression of the cytokine IL10 in the Angus cattle suggests a better immune, anti-inflammatory, and anti-oxidant system in this breed [36]; however, the upregulated expression of IL6, the pro-inflammatory cytokine, is puzzling. It is plausible that increased IL6 expression stimulates muscle growth, myogenesis, and energy metabolism [49,50], which could have resulted in better growth of the Angus breed; however, functional and mechanistic studies are needed to test this hypothesis. Although the underlying signaling pathways are not known at this time, the downregulated expression of IL10 by the TRT production system might be associated with the effect of RAC [51], despite the presence of the antibiotic tylosin phosphate, which has been reported to (not) affect circulating IL10 depending on the administered dose [52].

Similar to cytokines, the Angus breed seemed to have a greater circulating expression of chemokine ligands (CXCL14, CCL5, and CCL20) and CXC motif chemokine receptor 2 (CXCR2). The greater expression of the homeostatic, non-ELR (glutamic acid–leucine–arginine) chemokine CXCL14 suggests a better immune surveillance and antimicrobial immunity of the Angus breed [53], all of which can enhance growth. Although it has not been tested here, CXCL14 could modulate thermogenic adaptation by reducing energy expenditure and ameliorating growth in the Angus breed [54]. It is also probable that CXCL14 and CCL5 regulate glucose metabolism [55] and stimulate glucose uptake in the Angus breed [56,57]. In fact, CCL5 has been demonstrated to improve mitochondrial integrity, ATP production, and subsequent aerobic glucose metabolism [58], which might explain the greater growth rate of the Angus cattle. Moreover, CCL20 has been shown to improve muscle regeneration and growth through revascularization [59], which can be affected by HS [60,61].

Tight junction proteins are integral transmembrane proteins that function as both pores and barriers between cells, controlling the movement of fluids and solutes [62,63]. The upregulation of CLDN1 and OCLN expression in the whole blood of the Angus breed suggests that these genes might enhance blood cell integrity and reduce vessel leakiness and extravasation [64,65]. In addition, it is possible that these upregulated tight junction proteins were associated with a better immune system in the Angus cattle [66]. Functional tight junctions are also characterized by the linkage to the cytoskeleton that is accomplished by the protein family of membrane-associated guanylate kinase homologues (zonula occludens) and the adherens junction systems. Further studies are needed to assess the expression profile of these proteins. Most of these tight junction proteins are also involved in cellular signal transduction, and some as transcription factors under stress and pathological conditions, which also deserve further in-depth investigation in our research models.

In summary, this is the first report to the best of our knowledge showing that: (1) HSPs, (chemo)cytokines, and tight junction proteins are expressed in the whole blood of beef cattle, and (2) the expression of these genes is regulated in breed-, period-, and/or production system-dependent manners.

## Figures and Tables

**Table 1:** Oligonucleotide QPCR primers.

Gene ^1^	Accession Number ^2^	Primer Sequence (5′→3′)	Orientation	Product Size (bp)
*HSP60*	NM_001166609	CGCGGAAATGCTTCGATTAC	Forward	63
		GCCAGTGCCCTGGACACT	Reverse	
*HSPA1A*	NM_203322	GAGCTTCACGTCGTTGATCCT	Forward	59
		CGGCTCCGAGATAAGCTTCA	Reverse	
*HSP90*	NM_001012670	GCAAGATCGAACCCTCACCAT	Forward	59
		TCAAATCGGCCTTGGTCATC	Reverse	
*IL6*	NM_173923	GCCCTCCAGGAACAGCTATG	Forward	62
		GGAGACAGCGAATGGAGTGAA	Reverse	
*IL10*	NM_174088	GGCGGTGGAGAAGGTGAA	Forward	61
		GGCTTTGTAGACACCCCTCTCTT	Reverse	
*IL18*	NM_174091	ACAGTTCTGCTCTCCAATGCTTT	Forward	61
		GCCCCTTCAGCAGCAGAAG	Reverse	
*IL-1β*	NM_174093	GAGCCTGTCATCTTCGAAACG	Forward	55
		GCACGGGTGCGTCACA	Reverse	
*TNFα*	NM_173966	CGCATTGCAGTCTCCTACCA	Forward	56
		GGGCTCTTGATGGCAGACA	Reverse	
*CRP*	NM_001144097	TGGACATGAGTTTGAGCAAGCT	Forward	60
		CAGCACGCCAGGCTTTTC	Reverse	
*CCL2*	NM_174006	CCAAAGCCTTGAGCACTCACT	Forward	64
		AAGCCGGAAGAACACAAATTGT	Reverse	
*CCL4*	NM_001075147	TGCTCATGGCTGCCTTCTG	Forward	57
		GAGGGTCTGAGCCCATTGGT	Reverse	
*CCL5*	NM_175827	TTGCTTCTCGCTCTTGTCCTAA	Forward	59
		TGGGAGGAGGGCATTGC	Reverse	
*CCL20*	NM_174263	CCCAGTATTCTTGTGGGCTTCA	Forward	59
		GCATTGATGTCACAGGCTTCA	Reverse	
*XCL1*	NM_175716	AGCCAGGCCAAGCCTACAG	Forward	60
		CCCAGTCAGGGTCACAGTTGT	Reverse	
*CXCL12*	NM_001113174	AGATGCCCTTGCCGATTCT	Forward	56
		AGGTGCTTGACGTTGGCTTT	Reverse	
*CXCL14*	NM_001034410	CCGCTACAGCGACGTGAA	Forward	56
		CCTCGCAGTGCGGGTACTT	Reverse	
*CXCR1*	NM_174360	CCACCGTACTCCGACCTAGTCT	Forward	61
		TCCGCCATTTCGTTGTATTG	Reverse	
*CXCR2*	NM_001101285	CCGCCGCCCTTTCTTC	Forward	53
		TGTGGGACACCTCCAGGAA	Reverse	
*CCR2*	NM_001194959	CCACGTTCTTCCGAAAGCATA	Forward	62
		CCCATAGAAAACTGGGCATTG	Reverse	
*CLDN1*	NM_001001854	GCTCCTGTCCCCGGAAAA	Forward	61
		GGTGCTGGCTTGGGATAGG	Reverse	
*OCLN*	NM_001082433	GACTTCCGGCAGCCTCATTA	Forward	64
		CGGGAGCCCTTTTTGAAAG	Reverse	
*r18S*	NR_036642	CCGCGGTTCTATTTTGTTGGT	Forward	57
		CGGCCGCCCCTCTTAA	Reverse	

^1^ CCL, C-C motif chemokine ligand; CCR2, C-C motif chemokine receptor 2; CLDN1, claudin 1; CRP, C-reactive protein; CXCL, C-X-C motif chemokine ligand; CXCR, C-X-C motif chemokine receptors; HSP, heat shock protein; IL, interleukin; OCLN, occludin; TNFα, tumor necrosis factor alpha; XCL1, X-C motif chemokine ligand 1. ^2^ Accession number refers to Genbank (NCB).

**Table 2:** Effects of breed type and management system on body weight (Kg) of Angus and Brahman steers.

Breed (B)	Brahman (*Bos indicus*)		Angus (*Bos taurus*)					
Period (P) ^1^/System (S) ^2^	TRT	CON	TRT	CON	Three-Way ANOVA ^3^			
					S. of Variation	MS	F (DFn, DFd)	*p*
April (Initial)	346 ± 13 ^aα^	351 ± 16 ^aα^	338 ± 12 ^aα^	345 ± 6 ^aα^	P	179,786	86.22	<0.0001
May	383 ± 14 ^aα^	388 ± 18 ^aα^	391 ± 14 ^aα^	390 ± 8 ^aα^	B	56,831	27.25	<0.0001
June	412 ± 18 ^aα^	425 ± 21 ^aα^	462 ± 13 ^aβ^	443 ± 17 ^aβ^	S	10,006	4.798	0.0302
July	448 ± 19 ^aβ^	456 ± 26 ^aα^	508 ± 14 ^aβ^	493 ± 18 ^aβ^	P × B	4688	2.248	0.0424
August	483 ± 21 ^aβ^	460 ± 33 ^aα^	553 ± 17 ^aβ^	514 ± 18 ^aβ^	P × S	2602	1.248	0.2860
September	520 ± 22 ^aβ^	514 ± 34 ^aβ^	609 ± 21 ^aβ^	549 ± 17 ^aβ^	B × S	8482	4.068	0.0457
October (Final)	580 ± 23 ^aβ^	569 ± 38 ^aβ^	686 ± 18 ^bβ^	595 ± 23 ^aβ^	P × B × S	1116	0.5350	0.7808
					Residual	2085		

^1^ The study period was from April to October 2023. ^2^ CON, control; TRT, growth-promoting technology. ^3^ Three-way ANOVA showing individual and interaction effects of breed (B), production system (S), and period (P). Data are presented as mean ± SEM (n = 6 cattle/breed/system). For each month, means within a row with different superscript alphabetic letters are statistically different. For each group (breed and production system), means within a column with different superscript Greek letters are statistically different. All body weights are expressed using a 4% adjustment for rumen fill.

**Table 3:** Effects of breed and management system on daily growth rate of steers during summer season.

Breed (B)	Brahman (*Bos indicus*)		Angus (*Bos taurus*)					
Period (P) ^1^/System (S) ^2^	TRT	CON	TRT	CON	Three-Way ANOVA ^3^			
					S. of Variation	MS	F (DFn, DFd)	*p*
May	1.31 ± 0.1 ^aα^	1.29 ± 0.1 ^aα^	1.99 ± 0.1 ^aα^	1.68 ± 0.08 ^aα^	P	3.120	10.09	<0.0001
June	1.06 ± 0.1 ^aα^	1.24 ± 0.1 ^aα^	2.65 ± 0.1 ^bα^	2.05 ± 0.3 ^bα^	B	10.75	27.11	<0.0001
July	1.27 ± 0.09 ^aα^	1.16 ± 0.2 ^aα^	1.68 ± 0.07 ^aα^	1.86 ± 0.1 ^aα^	S	4.432	65.75	<0.0001
August	1.23 ± 0.1 ^aα^	0.29 ± 0.4 ^bβ^	1.57 ± 0.1 ^aα^	0.70 ± 0.2 ^cβ^	P × B	0.6586	4.458	0.0009
September	1.33 ± 0.1 ^aα^	1.71 ± 0.2 ^aα^	2.18 ± 0.2 ^bα^	1.35 ± 0.1 ^aα^	P × S	0.7288	4.029	0.0021
October	2.15 ± 0.06 ^aβ^	1.55 ± 0.1 ^bα^	2.63 ± 0.1 ^aα^	1.90 ± 0.3 ^abα^	B × S	1.021	6.248	0.0138
					P × B × S	0.4556	2.787	0.0203
					Residual	0.1635		

^1^ The study period was from April to October 2023. ^2^ CON, control; TRT, growth-promoting technology. ^3^ Three-way ANOVA showing individual and interaction effects of breed (B), production system (S), and period (P). Data are presented as mean ± SEM (n = 6 cattle/breed/system). For each month, means within a row with different superscript alphabetic letters are statistically different. For each group (breed and production system), means within a column with different superscript Greek letters are statistically different.

**Table 4:** Effects of breed and management system on HCW and dressing percent of steers during summer season.

Breed (B)	Brahman (*Bos indicus*)		Angus (*Bos taurus)*		Two-Way ANOVA (*p* Value) ^3^		
Parameter ^1^/System (S) ^2^	TRT	CON	TRT	CON	B	S	B × S
HCW (Kg)	368.8 ± 12.11	356.6 ± 9.18	430.9 ± 16.20	390.8 ± 8.87	0.0004	0.0314	0.2313
Dressing (%) ^4^	63.63 ± 0.92	64.83 ± 2.12	62.19 ± 3.13	63.13 ± 4.17	0.5880	0.7114	0.9641
	**Main Effect (Breed)**						
	**Brahman** (*Bos indicus*)		**Angus** (*Bos taurus*)		***t*-test** (*p* value)		
HCW (Kg)	362.7 ± 10.64		410.85 ± 12.53		0.0078		
	**Main Effect (Production System)**						
	**TRT**		**CON**		*p* value		
HCW (Kg)	399.85 ± 14.15		373.70 ± 9.02		0.1334		

^1^ HCW, hot carcass weight. ^2^ CON, control; TRT, growth-promoting technology. ^3^ Two-way ANOVA showing individual and interaction effects of breed (B) and production system (S). Data are presented as mean ± SEM (n = 6–12 cattle/breed/system). When the interaction was not significant, the main factors were analyzed separately using Student’s *t*-test. ^4^ Dressing % = (hot carcass weight/final BW) × 100.

## Data Availability

The data supporting the findings of the study are available within the article. Raw data supporting the findings are available from the corresponding author upon reasonable request.
